# Supplementary figures and images for: Design of a hesitant movement gesture for mobile robots
Source: PLoS One. 2021 Mar 25;16(3):e0249081. doi: 10.1371/journal.pone.0249081 (PMC7993606; doi:10.1371/journal.pone.0249081)

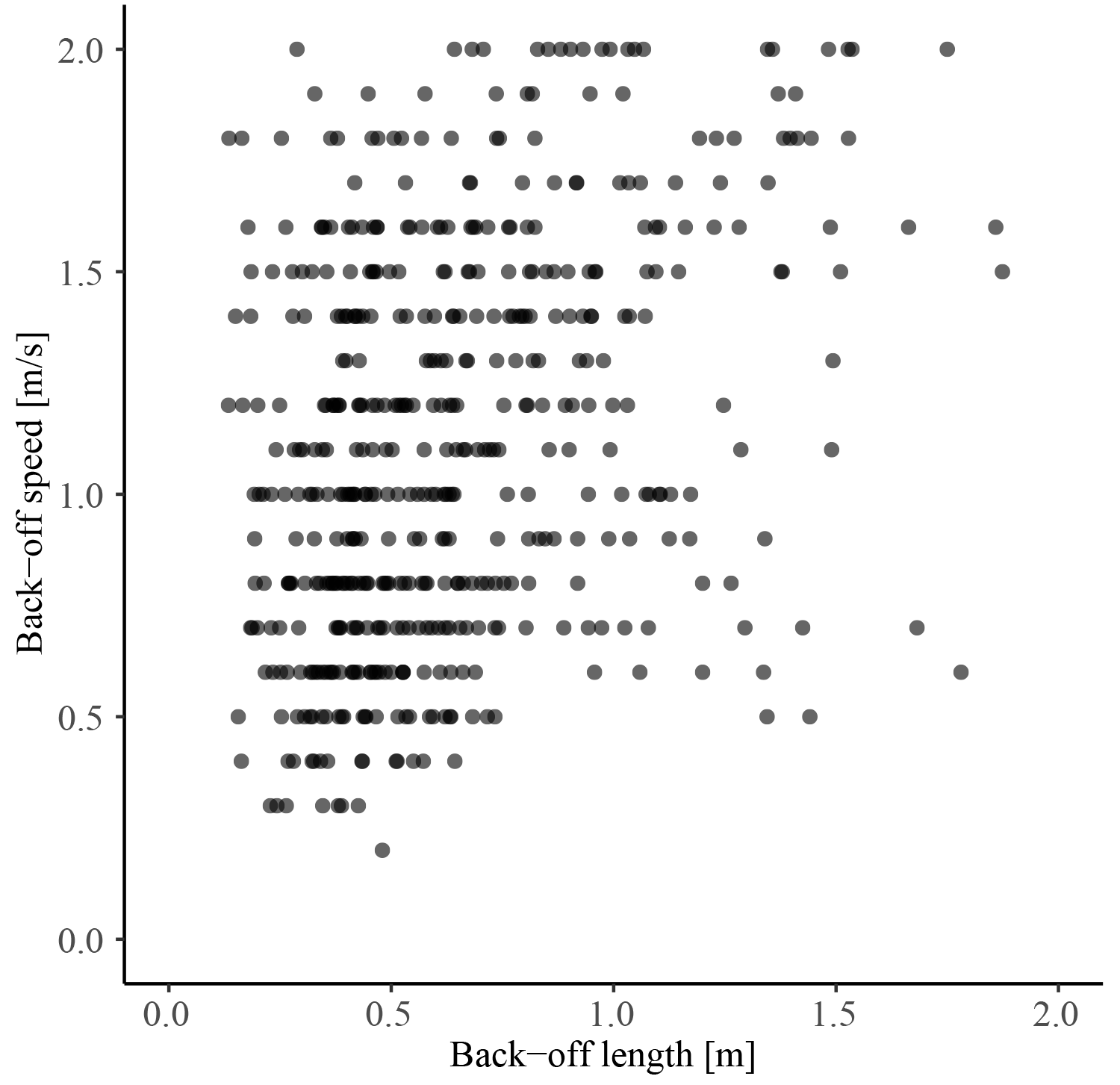

Supplement: S1 Fig — (TIF) [file pone.0249081.s001.tif]

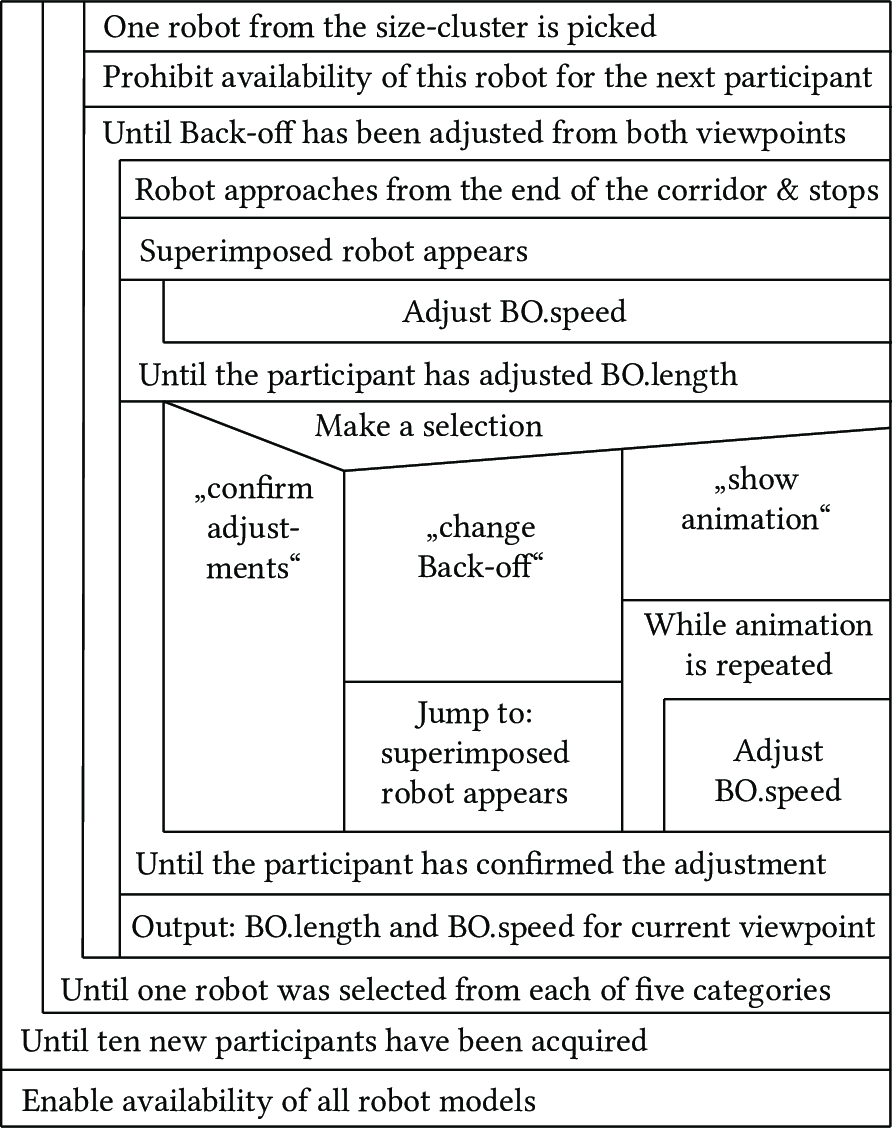

Supplement: S2 Fig — (TIF) [file pone.0249081.s002.tif]
