# Supplementary material for: Design of a hesitant movement gesture for mobile robots
Source: PLoS One. 2021 Mar 25;16(3):e0249081. doi: 10.1371/journal.pone.0249081 (PMC7993606; doi:10.1371/journal.pone.0249081)
Supplement: Attachment — Submitted filename: Response to reviewers.pdf [file pone.0249081.s005.pdf]

Technical University of Munich
Boltzmannstr. 15, 85747 Garching
Germany

December 18, 2020

Dear Prof. Dr. Quan Yuan,

Thank you very much for the review. As instructed, we have paid attention to the style requirements and used PACE tools to convert our images. Also, we adjusted the title page/heading according to the specifications you provided. Finally, we have made many changes to the manuscript. In the revised manuscript with track changes, deleted words and sentences are crossed out and marked red. Added words or sentences are highlighted in blue. In the following we would like to respond to the remarks raised by the reviewers. References to lines and chapters in the article point to the revised manuscript with track changes.

Remarks by Reviewer 1

This research considered the construction of human similarity of a robot. The method is proposed to make up a robots appearance and behavior that is perceived as human-like. However, the presentation and writing of the paper need to be substantially enhanced. Also, some key information and results should be explained in detail

Remark: *The presentation and writing of the paper must be substantially improved. The writing of current manuscript is hard to follow.*

Response: We improved the writing and language of the paper. For example, we found that "lateral" and "frontal" approach better explain the robot's travel directions. We dropped sentences that made the manuscript lengthy and improved the linkage of sentences as well as explanations. Also, the section on methodology has been restructured and the explanations of the experimental approach improved. We have divided additional sections of the text into paragraphs to support differentiation of the arguments.

Remark: *The highlight or important contribution of this paper is missing. Author needs to write the contributions of this paper clearly in section Introduction.*

Response: The important contribution of the paper is outlined in the introduction (line 15-25 of the revised manuscript with track changes). Our earlier work left open what combination of distance traveled, speed, execution time, and other properties such as the size of a robot will result in a harmonious back-off in the eyes of an observer. The aim is to meet the demand for a legible but also efficient movement strategy. Therefore, the movement must be sufficient to communicate the intention to yield priority and at the same time demand minimal path irregularities for a robot. Furthermore, to enable the use of back-off as a general motion language for robots, it would be necessary for developers to know how the design requirements for a back-off motion can be transferred to robots with different characteristics. The main contribution of this article is to provide a methodology for the tailoring of motion parameters to a variety of robots.

In the section on interpretation (Chapter 4.1) we demonstrate the application of the results of this paper with a calculation example. We use the linear-mixed model equation created to predict the required back-off length with robots of larger size (Equation 2) and insert the parameters defined by the model (Table 3). This would result in the following recommendation for the use of two new differently sized robots with a tailored behavior for interaction with a pedestrian from the lateral view: A robot, manufactured with double the size (0.5 m length increased to 1 m length) would need a back-off length increase from 0.606 m to 0.706 m to be legible.

Remark: *The presentation of the methodology in this paper is vague. The framework of section 2 needs to be improved.*

Response: The explanations and justifications for our methodology in Chapter 2 have been revised. In order to reduce repetition, keep the paper short and improve readability/explanations we combined the chapters "2.1 Study design and experimental conditions" and "2.2 Measures" into one chapter "2.1 Study design". As a consequence of this, the adjustments of the back-off length and back-off speed are explained in the same chapter as the explanations on how these parameters are applied to the robot behavior via Equation 1 (Chapter 2.1.4.). We believe that the combined explanation of measures and robot's behavior helps to improve clarity of the text. To add to this, the explanation of fixed and random effects (Chapter 2.1.3) is now before the explanation of robot behavior.

Furthermore, we have integrated the chapters "2.1.1 Virtual Environment" and "2.1.2 Virtual Reality - Technology" into one subsection "2.1.1 Virtual environment and technical configuration". We believe that the technology of use and the environment should be explained together.

Remark: *This paper is too long, and some key information is hard to be found. The reviewer suggests that the paper needs to be condensed and some key information needs to be highlighted.*

Response: We have shortened some parts that seemed lengthy and not entirely necessary for this article. In the revised manuscript with track changes, deleted words and sentences are crossed out and marked red. In the introduction some information from the referenced experiments is deleted. Some text passages are now divided into paragraphs to better high-

light some key information. We shortened the text by combining subsections and reducing repetitions. We were especially able to condense the manuscript in Chapters 1 and 2. Furthermore, we have highlighted key information such as the main motivations and contribution of this work. This is explained in more detail as an answer to the second remark.

Remarks by Reviewer 2

In this paper, a back-off movement strategy is designed to convey the robot intention to others. The study is interesting. There are some problems as follows.

Remark: *Why does this article choose a back-off movement strategy of robots to express the motion intention? Please explain it in details in Chapter 1.3. The innovative points of this article need to be further condensed and summarized.*

Response: This article chooses the back-off movement due to the fact that in our earlier work back-off was a promising means of using legible motion for nonverbal communication with humans. In particular, it proved to be better than stopping or rotating motion cues. Meanwhile, this accepted ACM THRI publication is published on a preprint server: (https://psyarxiv.com/ws2ez).

To fulfill the purpose of legibility for observers, a back-off movement has to be executed with a specific path to be perceivable and expressive. On the other hand, to be efficient and executable (time-loss, obstacles), such an evasive maneuver should be minimized. To allow the application in a variety of autonomous moving systems, our main research aim for this article is how appropriate back-off design can be modeled and translated to different sized robots and observer's viewpoints. In this article we take a closer look at the appropriate application of motion parameters that make the back-off movement legible. This was not done in such detail in our previous work. We adapted the statements in Chapter 1 (and Chapter 1.3. in particular) to further emphasize what the original work left open and how we continue in this article.

A condensed and illustrated way to describe the innovative points of this article can also be found in Chapter 4.1. We demonstrate the application of the main contribution of this paper by means of a calculation example. We use the linear-mixed model to predict the required back-off length for larger robots (cf. Equation 2) and insert the parameters defined by the model (cf. Table 3).

Remark: *The back-off movement proposed strategy lacks a detailed process of the method, an overall system scheme block diagram and flowchart in Chapter 2.*

Response: We improved the explanation of how the back-off maneuver is executed in chapter "2.1.4 Measures and robot behavior". A back-off maneuver is calculated based on the input of the two parameters set by a participant, length to cover and maximum speed (Equation. 1). The execution of speed as a function of time is associated with a certain amount of acceleration and deceleration during the maneuver. In order to create a realistic appearance of acceleration and deceleration, the speed is modeled with a cosine function.

In order to present the methodology and the procedure in an overall scheme, we have created

an additional system scheme block for the design of the back-off as part of the overall experimental procedure. This is done in the form of a Nassi-Shneiderman diagram and is provided as supplemental material (cf. S4 System). We have simplified Fig. 2, which now focuses on the selection of the robot model.

Remark: *Figure 3 and Figure 4 lack specific curve annotations.*

Response: We have added further curve annotations to Figures 3 and 4. The annotations describe that the lines represent linear equations resulting from the linear mixed model. The process of model generation is described in Chapters 3.1 and 3.2. We hope that we have now introduced the missing information content and that the comprehensibility is improved.

Kind regards,

Jakob Reinhardt
