# Supplementary material for: Design of a hesitant movement gesture for mobile robots
Source: PLoS One. 2021 Mar 25;16(3):e0249081. doi: 10.1371/journal.pone.0249081 (PMC7993606; doi:10.1371/journal.pone.0249081)
Supplement: Attachment — Submitted filename: Response to Reviewers.pdf [file pone.0249081.s006.pdf]

Technical University of Munich
Boltzmannstr. 15, 85747 Garching
Germany

February 18, 2021

Dear Prof. Dr. Quan Yuan,

Thank you very much for the review. In the revised manuscript with track changes, deleted words and sentences are crossed out and marked red. Added words or sentences are highlighted in blue. Reviewer 1 had no additional remarks. In the following we would like to respond to the remarks raised by the reviewer 2. References to lines and chapters in the article point to the revised manuscript with track changes.

Remarks by Reviewer 2

Remark:

1. It is recommended to add and condense the innovation points in the introduction and list them clearly. What specific method is used to design a hesitant movement gesture for mobile robots? What's the difference and innovation?

Response:

We condensed and listed the innovative points in the introduction (lines 25 - 28).

In the presented method, participants were directly involved in setting the movement parameters back-off length and back-off speed. The difference with previous research is that in the legible motion cue domain and our own previous study on back-off movements, predefined motion variations are typically used as independent variables in participant experiments (lines 11 - 16 and 204 - 207). These movements are designed by the experimenter. From this, information can typically be derived about which of the presented interaction strategies works best. However, research may overlook using the mental model and expectations of participants in the design process.

Such argumentation have been emphasized under the umbrella term "co-creation method"

(Sanders & Stappers, 2008) (line 207). In this article, we have presented an experimental approach that fully involves the participants in the design of the movement and allows for multiple design variations as a result.

Remark:

2. Why is execution time considered a promising parameter? Please give a clear explanation.

Response:

The execution time of a movement is a parameter that can be used to investigate speed and distance traveled in combination (line 408 - 421). If a participant changes the required back-off length and leaves the desired back-off speed at the same value, it means that with this setting he or she also changes the execution time of the maneuver. Similarly, if a participant changes the desired back-off speed while leaving the same required back-off length, he or she also changes the execution time. However, back-off length and back-off speed show a weak positive correlation in the dataset, which indicates a tendency that participants combined greater back-off lengths with higher speeds. It is possible that participants wanted to compensate greater back-off length with an increase in speed to keep the execution time at a similar level. Thus, the investigation of length and speed in combination may lead to a better understanding of the perception and expectations of motion. For this purpose we calculate an additional parameter composed by the speed of driving backwards over a certain length, the back-off time, in an exploratory analysis.

In the interpretation section of the article (lines 490 - 494) we argue that considering execution times of movement is in agreement with Lasseter (1987) who regard the timing of an action as one of the important principles in motion design. Additionally, in the studies of Knight & Simmons (2014) timing is one of the most important features separating the different paths.

Kind regards,

Jakob Reinhardt
